# Obstructive jaundice caused by a giant liver hemangioma with Kasabach-Merritt syndrome: a case report

**DOI:** 10.1186/s40792-015-0095-4

**Published:** 2015-10-06

**Authors:** Takuya Yano, Tsuyoshi Kobayashi, Shintaro Kuroda, Hironobu Amano, Hirotaka Tashiro, Hideki Ohdan

**Affiliations:** Department of Gastroenterological and Transplant Surgery, Applied Life Sciences, Institute of Biomedical & Health Sciences, Hiroshima University, 1-2-3 Kasumi, Minami-ku, Hiroshima, 734-8551 Japan

**Keywords:** Hemangioma, Obstructive jaundice, Biliary stricture, Kasabach-Merritt syndrome

## Abstract

Hemangioma is the most common benign tumor of the liver. Liver hemangioma (LH) usually remains asymptomatic, but the most common symptoms associated with LH are abdominal pain and discomfort. LH is an uncommon cause of bile duct dilatation and obstructive jaundice. An 83-year-old Japanese woman who received hemodialysis at another hospital was referred to our hospital because of abnormal liver function and obstructive jaundice. Abdominal computed tomography and magnetic resonance imaging revealed a 13-cm tumor in liver segments IV–V and intrahepatic bile duct dilatation. Endoscopic retrograde cholangiopancreatography revealed extrinsic compression of the bile duct at the hepatic hilar region. Laboratory tests showed that the patient had low platelet counts and low fibrinogen levels. Because the patient had hyperbilirubinemia and Kasabach-Merritt syndrome, we performed a segmentectomy of liver segments IV and V. Histological examination showed hemangioma of the liver. The patient’s thrombocytopenia and coagulopathy improved immediately after surgery. In conclusion, LH is a very rare cause of obstructive jaundice. LH has the potential to compress the bile duct and cause obstructive jaundice.

## Background

Liver hemangioma (LH) is the most common benign tumor arising in the liver, with an estimated prevalence of 0.4 to 7.3 % in the general population [1, 2]. Most LHs are reported to be asymptomatic. However, in patients undergoing surgery for hemangioma, 65 % of hemangiomas are symptomatic, with abdominal pain or discomfort being the most common symptoms [3]. The typical hemangioma is relatively small (<2 cm), but larger LH sometimes are observed, some measuring up to 30 cm in diameter. Biliary obstruction is a very rare manifestation of hemangioma.

We present a case with obstructive jaundice due to LH coexisting with Kasabach-Merritt syndrome, together with a discussion of the related medical literature.

## Case presentation

An 83-year-old woman with diabetes mellitus, hypertension, and spinal canal stenosis who was undergoing hemodialysis for chronic renal failure was admitted to another hospital, with itching as the chief complaint. She had notable hyperbilirubinemia (total bilirubin (T-Bil), 4.6 mg/dl; direct bilirubin (D-Bil), 3.7 mg/dl; alkaline phosphatase (ALP), 1333 U/L; gamma glutamyl transpeptidase (γ-GTP), 350 U/L) in blood examination. Abdominal echography examination revealed intrahepatic bile duct dilatation and computed tomography (CT) showed a 13-cm mass in the right lobe of the liver. Endoscopic retrograde cholangiopancreatography (ERCP) revealed extrinsic compression of the bile duct at the hepatic hilar region.

To improve the jaundice, endoscopic nasobiliary drainage was performed and yielded gradual improvements in the jaundice. At the same time, biliary cytology was performed. The architecture was disturbed, characterized by variation in internuclear distances and dyskaryosis. Therefore, the liver tumor was diagnosed as suspicious for malignancy. The patient was transferred to our hospital for hepatic tumor therapy.

Laboratory tests of biliary enzymes showed an improvement (T-Bil, 1.1 mg/dl; D-Bil, 0.6 mg/dl; ALP, 600 U/L; γ-GTP, 57 U/L) but revealed blood coagulopathy (platelet count, 9.6 × 10^3^ per cubic millimeter; fibrinogen, 122.5 mg/dl; fibrinogen and fibrin degradation products (FDP), 200 μg/ml; D-dimer, 88.8 μg/ml).

**Fig. 1:**
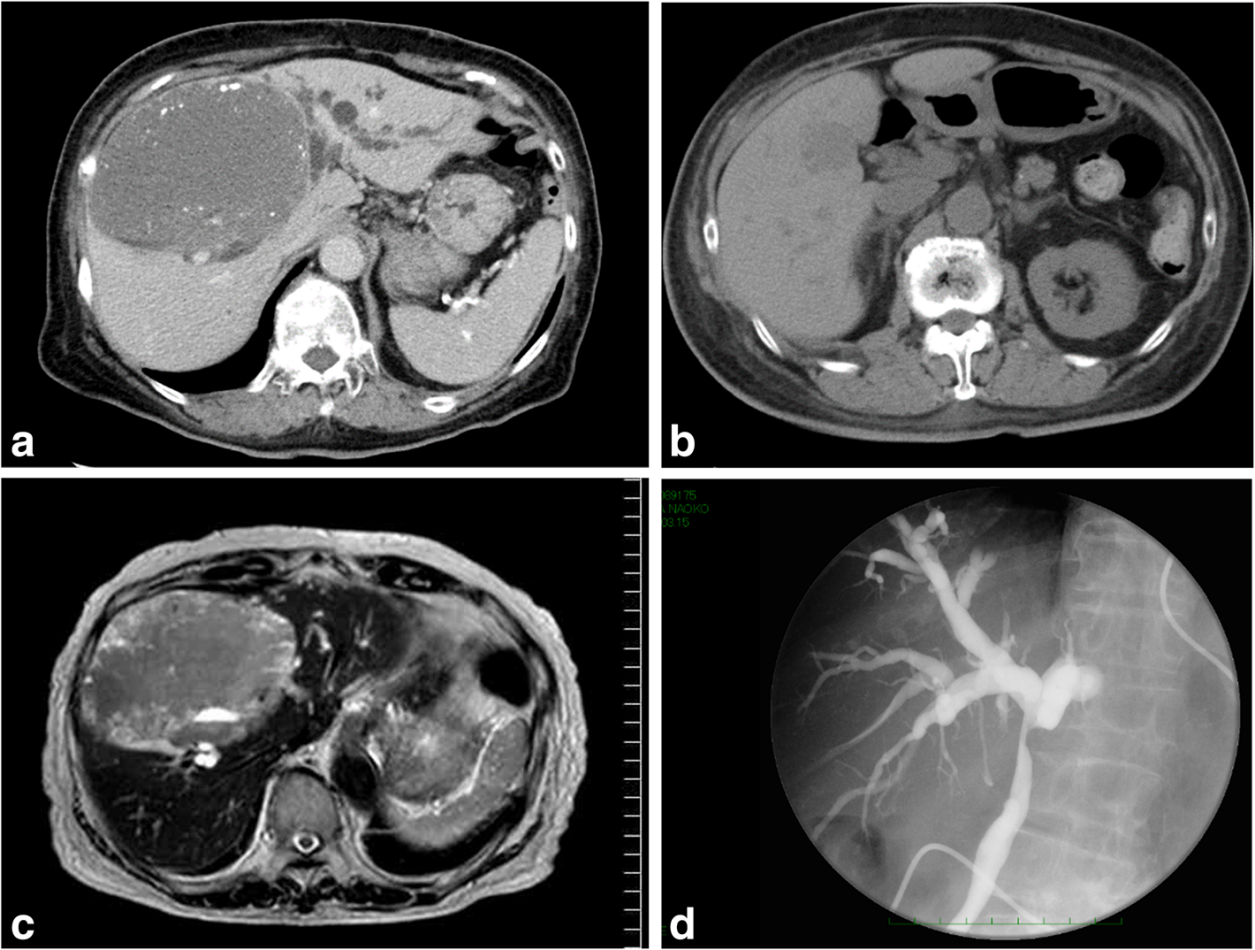
Radiological findings. **a** Computed tomography (CT) scan of the abdomen showing a well-demarcated liver mass consistent with hemangioma in the anterior segment of the right hepatic lobe to segment IV. The tumor pressed a bile duct in the hepatic portal region, causing an intrahepatic bile duct to expand. **b** A CT scan performed 5 years prior to the study shows a low-density tumor with a diameter of 28 mm in the right lobe. **c** The size of the tumor was shown to be 13 cm in diameter in magnetic resonance imaging, with a clearly demarcated border and a heterogeneous signal on T2-weighted images. The tumor had a fluid-fluid level at the tumor margins. **d** A stricture of approximately 2 cm in length in the common bile duct was observed via endoscopic retrograde cholangiography and suspected to be a result of compression from the outside wall. This caused partial expansion of a right lobe bile duct and high expansion of the left lobe bile duct

CT revealed a huge mass without enhancement, with a diameter of 13 cm, and clear boundaries in the anterior and medial sections of the liver. The tumor caused compression of a bile duct in the hepatic hilar region and intrahepatic bile ducts were dilated (Fig. 1a).

Previous CT scans 5 years ago at our hospital showed that the size of a tumor at the same locus was 28 mm (Fig. 1b).

A low signal on the T1-weighted image and a slightly high signal on the T2-weighted image were observed by using magnetic resonance imaging (MRI) (Fig. 1c).

A stricture of approximately 2 cm was observed in the common bile duct on ERCP and suspected to be a result of compression from the outside wall, causing partial expansion of a right lobe bile duct and high expansion of the left lobe bile duct (Fig. 1d).

The preoperative diagnosis was suspected to be cavernous hemangioma, but differential diagnosis suggested hemangioendothelioma and hepatic echinococcosis. Because of the malignant potential of the tumor and the coexistence of Kasabach-Merritt syndrome, surgery was performed.

Operative findings macroscopically showed a normal liver with a soft and multilocular 13-cm tumor localized in Segment IV–Segment V. A segmentectomy of the liver segments IV and V was performed with resection of the middle hepatic vein. We confirmed that a stricture of a bilateral hepatic duct was removed using intraoperative cholangiography. The intraoperative bleeding volume was 1930 ml. She received transfusion of 4 units of red blood cell transfusion, 5 units of fresh frozen plasma, and 10 units of platelet transfusion during the intraoperative period. She did not need transfusion before and after operation.

**Fig. 2:**
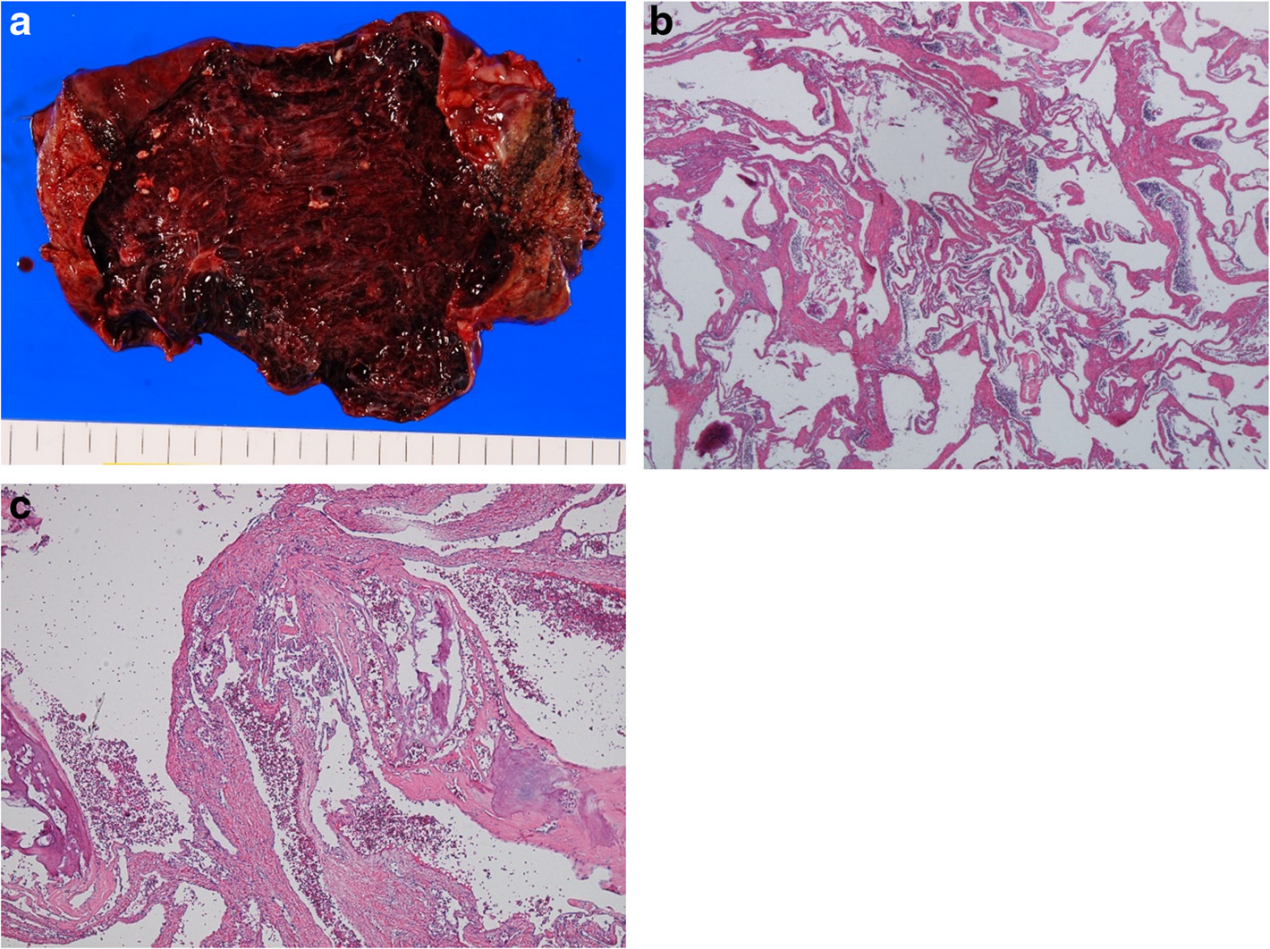
Gross and histological findings. **a** The tumor was wine colored and elastic. **b** The tumor was composed of blood-filled spaces lined by a single layer of endothelial cells without smooth muscle (H&E staining, objective; ×40). **c** Fibrotic, hyalinized, and calcified lesions were observed (H&E staining, objective; ×40)

Pathology revealed that the tumor was composed of cavernous vascular spaces of varying sizes lined by a single layer of flat endothelium. There was focal stromal fibrosis, calcification, and hyalinization (Fig. 2). Our final diagnosis of the patient was cavernous hemangioma of the liver.

**Fig. 3:**
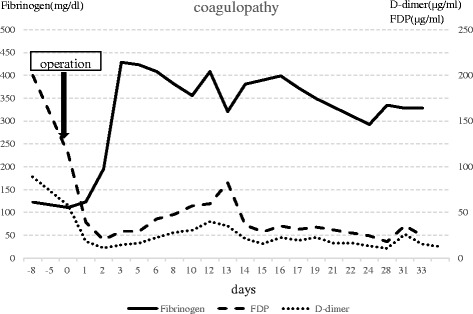
Clinical course of coagulopathy. After surgery, blood coagulopathy was improved immediately

Postoperative cholangiography did not show a biliary stricture. After surgical resection of the hemangioma, blood coagulopathy improved (Fig. 3) (platelet count, 183 per cubic millimeter; fibrinogen, 328.1 mg/dl, FDP, 24.4 μg/ml; D-dimer, 12.6 μg/ml), and the patient was able to resolve her thrombocytopenia and coagulopathy.

### Discussion

Most patients with cavernous hemangioma are asymptomatic but a minority present with abdominal pain or abdominal distension. Hemangiomas have been identified at autopsy in up to 7 % of patients and are more common in women than in men [1]. Lesions are usually dark red and well circumscribed, but the macroscopic appearance can vary because of thrombosis, fibrosis, and calcification. Histologically, cavernous hemangiomas are composed of blood-filled spaces lined by a single layer of endothelial cells and supported by a basement membrane. Fibrosis is variable but may involve the entire lesion (sclerosed hemangioma). Rare complications include spontaneous rupture, traumatic rupture, portal hypertension, and coagulopathy. The identification of hemangiomas using radiological imaging can be difficult, but investigations with relatively high sensitivity and specificity include MRI scans, contrast-enhanced CT, and nuclear medicine scans using labeled red cells.

Dilatation of the intrahepatic bile ducts can occur as a result of either compression or tumor spread in patients with malignant liver tumor, such as (1) tumor spread to and sheathing of the bile ducts in cholangiocarcinomas, (2) growing cast within the bile ducts in hepatocellular carcinomas, and (3) metastatic infiltration of the bile ducts, particularly in colon adenocarcinoma [4]. Bile duct dilatation can be an important indicator of malignant liver tumors. In contrast, benign liver tumors are not considered to be a cause of bile duct dilatation, even if they are very large [5, 6].

Until now, only two resected adult cases with obstructive jaundice by cavernous hemangioma have been reported in the English literature (Table 1) [7, 8]. Including this case, all cases were observed in women with an age of 31–83 years. The tumor was 13–20 cm in diameter. One report was of a biliary stricture caused by a hepatic cyst [6]. The locus of the hepatic cysts was an existing case close to the hilum. Even in benign cases, other structures may show the presence of biliary stricture in lesions close to the hilum [9]. Two hemangioma cases causing the biliary stricture were close to the hilum similarly. In our case, the tumor was located in segment IV, close to the hilum. If hemangioma is close to the hilum, there is a possibility that hemangioma can cause biliary stricture regardless of tumor size.

**Table 1:** Reported cases of liver hemangioma causing biliary stricture

Year	First author	Age	Sex	Symptom	Laboratory data	Location	Tumor size	Operation
2008	Julian E. Losanoff	42	F	Discomfort in the right hypochondrium and itching	T-Bil 4.2 mg/dl, D-Bil 3.9 mg/dl, ALP 283 IU/L	Right lobe	15 cm	Right hepatectomy
2009	L Tang	31	F	Jaundice and amenorrhea	T-Bil 292 μmol/l, ALP 575U/l	–	20 cm	Right hepatectomy
2015	Present	83	F	Itching	T-Bil 4.6 mg/dl, D-Bil 3.7 mg/dl, ALP 1333 IU/L	S4-S5	13 cm	Segmentectomy

*T-Bil* total birilubine, *D-Bil* direct birilubine, *ALP* alkaline phosphatase

The incidence of Kasabach-Merritt syndrome in LH is around 1.7 % [3], but Kasabach-Merritt syndrome is a serious complication characterized by a very large hemangioma with thrombocytopenia and consumption coagulopathy due to endothelial defects within the hemangioma. In our case, according to CT, the hemangioma had enlarged over 5 years. The enlarging of the hemangioma from 5 years ago caused Kasabach-Meritt syndrome, and the hemangioma, to reach hepatic portal region. We consider that the hemangioma which was close to hilum induced bile duct stricture. There is another speculation about Kasabach-Meritt syndrome and bile duct stricture. Consumptive coagulopathy due to Kasabach-Meritt syndrome might cause blood flow disorders of bile duct. But there were no reports of a hemangioma with Kasabach-Merritt syndrome as the cause for biliary stricture and development of obstructive jaundice.

There are some reports that estrogen contributes to hemangioma enlargement [10, 11]. Our case occurred after menopause in the absence of estrogen intake. However, the patient received oral prostaglandin E1, which contains a platelet aggregation inhibitor, to treat spinal canal stenosis and underwent hemodialysis. We suspected that the patient developed Kasabach-Merritt syndrome due to the enlargement of tumor with tendency to bleed.

LHs that are large and symptomatic are usually treated surgically, either by enucleation or by resection. LHs require some form of treatment, such as surgical resection, liver transplantation [12–14], arterial embolization [15, 16], interferon [17, 18], radiation [19], systemic corticosteroids [20], or radiofrequency ablation [21]. In general, hepatic hemangiomas with Kasabach-Merritt syndrome should be managed by surgical approaches if resection is possible [22]. As there are no reports on the use of transcatheter arterial embolization therapy or bile duct stents in cases where the hemangioma causes biliary stricture, as in this case, surgical resection is currently considered an effective treatment.

## Conclusions

LHs may be enlarged, and those close to the hepatic hilum may potentially compress the bile duct and cause obstructive jaundice. Therefore, there is a need to carefully observe hemangiomas.

## Consent

Written informed consent was obtained from the patient for publication of this case report and any accompanying images. A copy of the written consent is available for review by the Editor-in-Chief of this journal.

## Abbreviations

LH: liver hemangioma
T-Bil: total bilirubin
D-Bil: direct bilirubin
ALP: alkaline phosphatase
γ-GTP: gamma glutamyl transpeptidase
CT: computed tomography
ERCP: endoscopic retrograde cholangiopancreatography
MRI: magnetic resonance imaging
